# The Global Decline in Sperm Count and Testosterone Levels: Trends, Mechanisms, and Environmental Drivers

**DOI:** 10.3390/antiox15060778

**Published:** 2026-06-22

**Authors:** Sandro La Vignera, Rosita A. Condorelli

**Affiliations:** Department of Clinical and Experimental Medicine, University of Catania, 95123 Catania, Italy; rosita.condorelli@unict.it

**Keywords:** sperm count decline, testosterone, endocrine disruptors, male infertility, hypothalamic–pituitary–gonadal axis, oxidative stress, bisphenol A, phthalates, antioxidant therapy

## Abstract

Male reproductive health has experienced an unprecedented decline over the past five decades, characterized by substantial reductions in sperm count and testosterone levels. This review provides a comprehensive synthesis of current evidence on the global decline in sperm count and testosterone levels, examining epidemiological trends, underlying mechanisms, environmental drivers, and clinical implications. Sperm concentration declined by 51.6% globally between 1973 and 2018, with an accelerating trajectory post-2000 (from 1.16% to 2.64% per year). Concurrently, multiple independent studies document an age-independent secular decline in testosterone, averaging 1–2% per year across diverse populations. The etiology is multifactorial, involving endocrine-disrupting chemicals (bisphenol A, phthalates, pesticides, dioxins), lifestyle factors (obesity, sedentary behavior, smoking, heat exposure), and disruption of the hypothalamic–pituitary–gonadal axis. At the cellular level, mechanisms include Sertoli and Leydig cell dysfunction, oxidative stress, mitochondrial impairment, and sperm DNA fragmentation. Integrated clinical management combining lifestyle optimization, antioxidant therapy, and targeted endocrine interventions is essential. Prevention through environmental policy and public health initiatives represents the most promising long-term strategy.

## 1. Introduction

Male reproductive health has emerged as a critical public health concern in the early twenty-first century, with accumulating evidence documenting substantial declines in sperm count and testosterone levels across diverse populations worldwide. This phenomenon, often termed the “male infertility crisis,” represents one of the most significant and underappreciated threats to human reproductive capacity and overall health [1,2]. The implications extend beyond fertility, encompassing metabolic dysfunction, cardiovascular disease, bone health, psychological well-being, and potentially population-level demographic shifts [3,4].

The recognition of declining sperm counts began with the seminal work of Carlsen and colleagues in 1992, who reported a nearly 50% decrease in mean sperm concentration from 113 million/mL in 1940 to 66 million/mL in 1990 based on a systematic review of 61 studies [5]. This provocative finding sparked intense scientific debate and subsequent investigations that have largely confirmed and extended these observations [6,7]. More recently, comprehensive meta-analyses by Levine and colleagues have provided robust evidence for a continuing and accelerating decline in sperm parameters, particularly among men from Western countries [2,8].

Concurrently, multiple independent studies have documented an age-independent secular decline in testosterone levels, with population-level decreases of approximately 1% per year observed in American, Danish, Finnish, and Israeli populations [9,10,11,12]. This decline occurs independently of aging and is not fully explained by increases in obesity or other measured health factors, suggesting the involvement of environmental or lifestyle changes affecting entire populations [9,13].

The significance of declining male reproductive parameters extends to population-level fertility dynamics. Global total fertility rates (TFRs) have fallen dramatically, from 4.84 births per woman in 1950 to 2.23 in 2021, with projections indicating a further decline to 1.83 by 2050 and 1.59 by 2100 [14,15]. While socioeconomic factors—female education, urbanization, and reduced infant mortality—are primary drivers of this demographic transition, emerging evidence suggests that declining biological fecundity contributes an additional, underappreciated component. Infertility affects approximately 15% of couples worldwide, with male factors contributing to roughly half of all cases [14,16]. Recent analyses document declining unassisted pregnancy rates in high-income countries, suggesting that reduced biological fecundity—including impaired male reproductive function—may be compounding the behavioral and socioeconomic drivers of falling birth rates [Lindahl-Jacobsen et al., Fertil Steril, 2025; Skakkebæk et al., Nat Rev Endocrinol, 2021 [16,17]. Understanding the interplay between male reproductive decline and global fertility trends is therefore critical for both clinical practice and public health policy.

The etiology of this dual decline in male reproductive parameters is multifactorial and complex. Endocrine-disrupting chemicals (EDCs)—ubiquitous environmental contaminants including bisphenol A (BPA), phthalates, pesticides, dioxins, and polychlorinated biphenyls (PCBs)—have emerged as prime suspects [18,19,20]. These compounds interfere with hormone synthesis, receptor binding, and cellular signaling pathways critical for spermatogenesis and steroidogenesis [21,22]. Additionally, lifestyle factors including obesity, sedentary behavior, smoking, alcohol consumption, heat exposure, and psychological stress contribute synergistically to reproductive dysfunction [23,24,25].

At the cellular and molecular level, the mechanisms underlying male reproductive decline involve disruption of the hypothalamic–pituitary–gonadal (HPG) axis, Leydig cell senescence and dysfunction, Sertoli cell impairment, oxidative stress, mitochondrial dysfunction, DNA damage, and epigenetic modifications [26,27,28]. These pathophysiological processes converge to impair spermatogenesis, reduce testosterone production, and compromise sperm quality and function [29,30].

**Figure 1:**
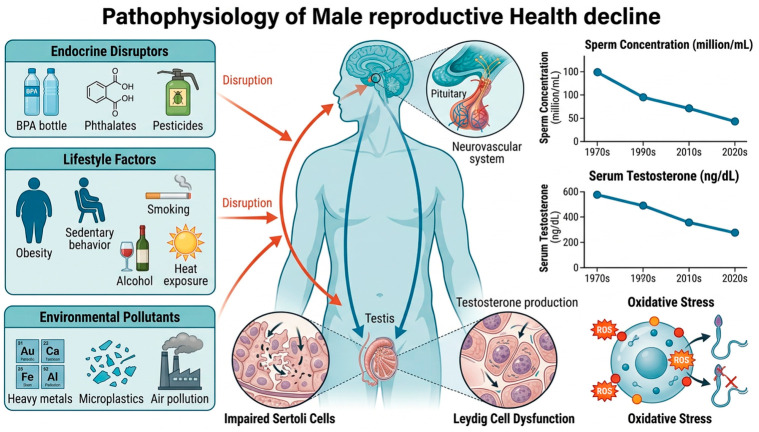
Integrated pathophysiology of male reproductive health decline. The figure illustrates the multifactorial etiology of declining sperm count and testosterone levels, including environmental exposures (endocrine-disrupting chemicals, heavy metals, air pollution), lifestyle factors (obesity, smoking, sedentary behavior), and their convergent effects on the HPG axis, Sertoli cells, Leydig cells, and oxidative stress pathways. This figure was created with the assistance of artificial intelligence (AI) tools (ChatGPT-4, OpenAI, San Francisco, CA, USA). The AI-generated illustration was reviewed and approved by the authors.

This review provides a comprehensive synthesis of current evidence on the global decline in sperm count and testosterone levels, examining epidemiological trends, underlying mechanisms, environmental drivers, and clinical implications. We emphasize the urgent need for integrated approaches combining environmental policy, lifestyle modification, and targeted medical interventions to address this escalating public health crisis (Figure 1).

## 2. Global Epidemiological Trends in Sperm Count

### 2.1. The Levine Meta-Analyses: Defining the Crisis

The most comprehensive and methodologically rigorous assessment of temporal trends in sperm count comes from the systematic reviews and meta-regression analyses conducted by Levine and colleagues. The 2017 analysis examined 185 studies, including 42,935 men who provided semen samples between 1973 and 2011 [2]. This landmark study demonstrated a significant decline in sperm concentration (SC) among “Unselected Western” men (from North America, Europe, Australia, and New Zealand) of 1.4% per year, resulting in a total decline of 52.4% over the study period. Total sperm count (TSC) declined even more dramatically at 1.6% per year, representing a 59.3% overall decrease [2].

Critically, these declines were observed specifically among men unselected by fertility status, suggesting a population-level phenomenon rather than changes in the composition of men seeking fertility evaluation. The meta-regression analysis controlled for potential confounders, including abstinence time, age, and geographic location, strengthening the validity of these findings [2].

The updated 2022 meta-analysis extended these observations through 2018 and expanded geographic coverage to include data from South America, Asia, and Africa [8]. This analysis of 223 studies with 288 estimates confirmed the continuing decline in sperm concentration, which reached 51.6% among unselected men globally between 1973 and 2018 [8]. The slope of decline was −1.17 million/mL per year (*p* < 0.001). Importantly, the rate of decline appeared to accelerate post-2000, increasing from 1.16% per year pre-2000 to 2.64% per year after 2000 [8].

For the first time, significant declines were also documented in men from South/Central America, Asia, and Africa, with a slope of −0.65 million/mL per year (*p* = 0.045), though the magnitude was smaller than in Western countries [8]. Total sperm count declined by 62.3% overall, confirming the robustness of this trend across multiple semen parameters [8].

### 2.2. Geographic Variations and Regional Patterns

While the Levine meta-analyses demonstrate global trends, substantial geographic heterogeneity exists in both baseline sperm parameters and temporal trends. European studies have consistently reported lower sperm concentrations compared to other regions, with particularly pronounced declines in Denmark, France, and Scotland [31,32]. A large Parisian study of 1351 healthy men revealed a yearly decrease of 2.6% in sperm concentration, representing one of the steepest documented declines [33].

In contrast, some regions have shown stable or even increasing sperm counts. A systematic review of Chinese men, including 327,373 individuals from 1981 to 2019, found complex temporal patterns that varied by region and time period [34]. Similarly, a study of fertile men from 10 countries across four continents found no significant temporal trends in sperm concentration or total sperm count between 2010 and 2021, though this analysis was limited to men with proven paternity [35].

These geographic variations likely reflect differences in environmental exposures, lifestyle factors, genetic backgrounds, and potentially methodological differences in semen analysis [36,37]. Regional differences in exposure to endocrine-disrupting chemicals, air pollution, dietary patterns, and occupational hazards may contribute to the observed heterogeneity [38,39].

### 2.3. Temporal Acceleration and Recent Trends

A particularly concerning finding from recent analyses is the apparent acceleration of sperm count decline in the twenty-first century. The 2022 Levine meta-analysis documented that the rate of decline more than doubled after 2000, increasing from 1.16% to 2.64% per year [8]. This acceleration suggests that the factors driving sperm count decline are intensifying rather than stabilizing.

Several hypotheses may explain this acceleration. First, exposure to endocrine-disrupting chemicals has increased substantially in recent decades, with production and use of plastics, pesticides, and industrial chemicals expanding globally [40,41]. Second, the obesity epidemic has accelerated since the 1990s, with metabolic dysfunction now affecting a substantial proportion of reproductive-age men [42]. Third, lifestyle factors including sedentary behavior, screen time, sleep deprivation, and psychological stress have intensified in modern societies [43,44].

**Figure 2:**
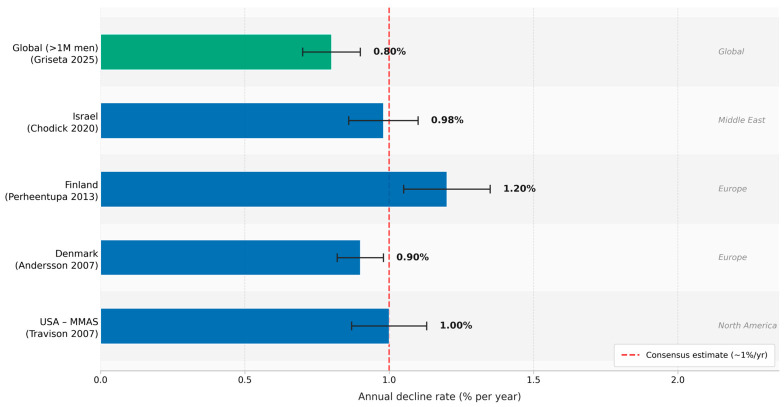
Secular decline in serum testosterone levels by population. Percentage decline per year by geographic region based on meta-regression analyses. Comparison of sperm concentration trajectories across major world regions from 1973 to 2018. Data derived from: Levine et al. (2017) [2]; Levine et al. (2022) [8]; Pasqualotto et al. (2003) [29]; Lv et al. (2021) [30]; Cannarella et al. (2023) [31]. Error bars represent reported ranges or confidence intervals [10,11,12,47,48].

The clinical significance of these trends is substantial. Mean sperm concentration in Western men has declined from approximately 99 million/mL in 1973 to 49 million/mL in 2018 [8]. While this remains above the World Health Organization (WHO) reference limit of 16 million/mL (5th percentile), the population distribution has shifted such that an increasing proportion of men now fall below clinical thresholds for optimal fertility [45]. Moreover, the relationship between sperm concentration and fertility is continuous rather than threshold-based, meaning that declines even within the “normal” range may impair reproductive capacity at the population level [46] (Figure 2).

## 3. Testosterone Decline: An Age-Independent Secular Trend

### 3.1. Population-Level Evidence

Parallel to the decline in sperm count, multiple independent studies have documented a population-level decrease in testosterone levels that is independent of aging. The first clear demonstration of this phenomenon came from the Massachusetts Male Aging Study (MMAS), where Travison and colleagues reported a substantial age-independent decline in serum testosterone levels in American men between 1987–1989 and 2002–2004 [9]. This decline persisted after adjusting for age, body mass index (BMI), smoking status, and health conditions, suggesting a true secular trend affecting the entire population [9].

Subsequent studies have confirmed this finding across diverse populations. In Denmark, analysis of 5350 male serum samples from four population surveys conducted between 1982 and 2001 revealed an age-independent decrease in testosterone levels accompanied by an increase in sex hormone-binding globulin (SHBG) [10]. A Finnish cohort study demonstrated a clear birth cohort effect, with more recently born men having lower testosterone levels than earlier cohorts at the same age [11]. For example, men aged 60–69 born between 1942 and 1951 had a mean testosterone of 13.8 nmol/L, compared to 21.9 nmol/L for those born between 1913 and 1922 [11].

More recent data from Israel, analyzing testosterone levels in men between 2006 and 2019, showed a significant and prominent age-independent decline [12]. At age 21, testosterone levels declined from 19.68 nmol/L in 2006–2009 to 17.76 nmol/L in 2016–2019 (*p* < 0.001) [12]. Importantly, this decline was unlikely to be explained by increasing obesity rates, as the trend persisted after adjustment for BMI [12].

A large-scale analysis of over one million healthy men revealed a significant negative linear regression between serum testosterone levels and year of measurement (coefficient −0.6, SE: 0.2, *p* < 0.001), independent of age and BMI [47]. Notably, luteinizing hormone (LH) levels also declined in parallel (coefficient −0.1, SE: 0.1, *p* < 0.001), suggesting an ongoing resetting of the hypothalamic-pituitary-testicular axis rather than isolated testicular failure [47].

### 3.2. Longitudinal Versus Cross-Sectional Studies

An important methodological consideration in assessing testosterone decline is the distinction between cross-sectional and longitudinal studies. Cross-sectional studies comparing different age groups at a single time point typically show testosterone declining by approximately 0.3–1.0% per year with aging [49,50]. However, longitudinal studies following the same individuals over time reveal steeper declines of 1.5–2.6% per year [48,51,52].

This discrepancy suggests that secular trends contribute substantially to the observed age-related decline in testosterone. The Baltimore Longitudinal Study of Aging found independent, age-invariant declines in total testosterone of −0.124 nmol/L per year and free testosterone index of −0.0049 nmol T/nmol SHBG per year [49]. A study of elderly men over 70 years showed bioavailable testosterone declining by 6.1 ng/dL per year, independent of baseline age [50].

The magnitude of the secular decline is substantial. Longitudinal analysis of men in the MMAS showed mean testosterone fell by 207 ng/dL over 20 years, a decline twice what cross-sectional estimates predicted for aging alone [13]. An age-independent secular decline accounted for 178 ng/dL of this loss [13]. Remarkably, even men who maintained stable weight experienced a 117 ng/dL (19%) testosterone reduction, indicating that obesity alone cannot explain the secular trend [13].

### 3.3. Clinical Implications of Hypogonadism

The population-level decline in testosterone has significant clinical implications. The prevalence of biochemical hypogonadism (typically defined as total testosterone < 300 ng/dL or <10.4 nmol/L) increases substantially with age, affecting approximately 20% of men over 60, 30% over 70, and 50% over 80 years [49,53]. However, the secular decline means that younger men today have testosterone levels comparable to older men from previous generations [11,12].

Low testosterone is associated with a constellation of adverse health outcomes beyond sexual dysfunction. Epidemiological studies have linked hypogonadism to increased risks of type 2 diabetes, metabolic syndrome, cardiovascular disease, osteoporosis, sarcopenia, cognitive decline, depression, and all-cause mortality [54,55,56]. The relationship appears bidirectional, with low testosterone contributing to metabolic dysfunction and chronic diseases, while metabolic disorders further suppress testosterone production [27,28].

Sexual symptoms associated with low testosterone include reduced libido, erectile dysfunction, and decreased spontaneous erections [55]. Non-sexual symptoms encompass fatigue, reduced muscle mass and strength, increased body fat, decreased bone mineral density, mood disturbances, and cognitive impairment [57,58]. However, the relationship between testosterone levels and symptoms is complex and variable, with substantial individual differences in sensitivity to androgen deficiency [59].

**Figure 3:**
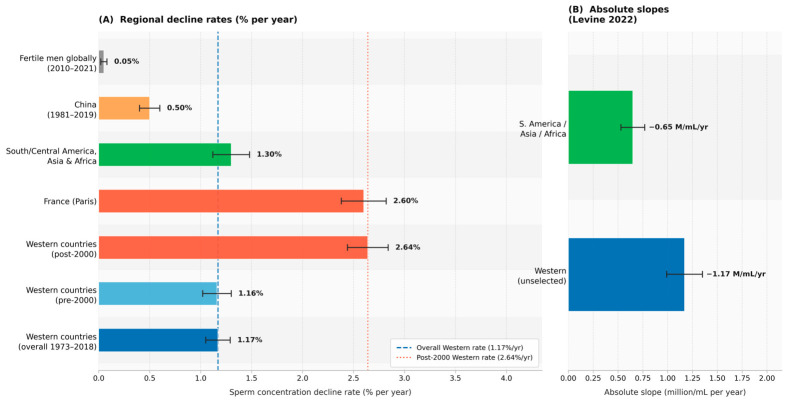
Regional trends in sperm concentration decline. Horizontal bars show the age-independent rate of decline (% per year) reported in major longitudinal and cross-sectional studies. Data derived from: Travison et al. (2007) [9]; Andersson et al. (2007) [10]; Perheentupa et al. (2013) [11]; Chodick et al. (2020) [12]; Griseta et al. (2025) [43]. Error bars represent reported ranges or confidence intervals [8].

The clinical threshold for testosterone deficiency remains debated, with various guidelines proposing cutoffs ranging from 230 to 350 ng/dL (8–12 nmol/L) [53,54]. Free or bioavailable testosterone may be more clinically relevant than total testosterone, particularly in older men with elevated SHBG [60]. The diagnosis of late-onset hypogonadism requires both biochemical evidence (consistently low testosterone on morning samples) and clinical symptoms [61] (Figure 3).

## 4. Mechanisms of Spermatogenic Impairment

### 4.1. Hypothalamic–Pituitary–Gonadal Axis Disruption

The hypothalamic–pituitary–gonadal (HPG) axis represents the central regulatory system controlling male reproductive function. Disruption of this axis at any level can have profound effects associated with impaired spermatogenesis and testosterone production. The axis operates through a negative feedback loop: hypothalamic gonadotropin-releasing hormone (GnRH) stimulates pituitary secretion of luteinizing hormone (LH) and follicle-stimulating hormone (FSH), which in turn stimulate testicular Leydig cells (testosterone production) and Sertoli cells (spermatogenesis support), respectively [4].

Multiple environmental and lifestyle factors have been associated with disruption of HPG axis function. Obesity, associated with hyperestrogenism, resulting from aromatization of androgens to estrogens in adipose tissue, suppresses GnRH and gonadotropin secretion, leading to secondary hypogonadism [27,28]. Chronic inflammation, mediated by pro-inflammatory cytokines such as interleukin-6 (IL-6) and tumor necrosis factor-alpha (TNF-α), directly inhibits hypothalamic GnRH neurons and pituitary gonadotrophs [54,56].

Endocrine-disrupting chemicals can interfere with HPG axis function through multiple mechanisms. Some EDCs exhibit estrogenic activity, activating estrogen receptors and mimicking the negative feedback effects of estradiol on GnRH and gonadotropin secretion [20,21]. Others possess anti-androgenic properties, blocking androgen receptors and disrupting the normal feedback regulation [19]. Dioxins and related compounds act through the aryl hydrocarbon receptor (AhR), which can interfere with steroid hormone signaling and reproductive axis function [62].

Aging is associated with alterations in HPG axis function, including decreased GnRH pulse frequency and amplitude, blunted LH response to GnRH stimulation, and increased sensitivity of the hypothalamus and pituitary to testosterone feedback [29,58]. However, the secular decline in both testosterone and LH levels observed in recent studies suggests a population-level resetting of the HPG axis beyond normal aging [47].

### 4.2. Sertoli Cell Dysfunction

Sertoli cells are the somatic cells of the seminiferous epithelium that provide structural, nutritional, and regulatory support for developing germ cells. Each Sertoli cell can support a finite number of germ cells, and the number of Sertoli cells, established during fetal and early postnatal life, determines the maximum sperm production capacity of the testis [18,62]. Sertoli cell dysfunction represents a critical mechanism underlying impaired spermatogenesis.

Environmental exposures during fetal development can permanently reduce Sertoli cell number and function, a concept central to the testicular dysgenesis syndrome (TDS) hypothesis [21,62]. Maternal exposure to endocrine disruptors, particularly anti-androgenic compounds, during the critical masculinization programming window has been associated with impaired Sertoli cell proliferation and differentiation [63]. This results in reduced adult Sertoli cell number, decreased sperm production capacity, and increased risk of cryptorchidism, hypospadias, and testicular cancer [18].

In adulthood, Sertoli cells remain vulnerable to environmental insults. Phthalates, particularly di-(2-ethylhexyl) phthalate (DEHP), have been associated with impaired Sertoli cell function by disrupting tight junction proteins, impairing the blood–testis barrier, and reducing production of growth factors and hormones essential for germ cell development [20,64]. Heavy metals such as cadmium have been associated with disruption of inter-Sertoli cell tight junctions, compromising the immune-privileged environment of the seminiferous tubules [62].

Oxidative stress represents another major threat to Sertoli cell function. Sertoli cells possess antioxidant defense systems that protect developing germ cells from reactive oxygen species (ROS), but excessive oxidative stress can overwhelm these defenses [20]. Environmental toxicants, heat exposure, and metabolic dysfunction can all be associated with oxidative stress in Sertoli cells, impairing their support function and leading to germ cell apoptosis [19,62].

FSH signaling is essential for Sertoli cell function, regulating expression of genes involved in germ cell support, metabolism, and cell–cell communication [4]. Disruption of FSH signaling, whether through HPG axis suppression or direct interference with FSH receptors, compromises Sertoli cell function and spermatogenesis [21].

### 4.3. Leydig Cell Impairment and Steroidogenic Failure

Leydig cells, the testosterone-producing cells of the testis, are critical for both spermatogenesis (through high intratesticular testosterone concentrations) and systemic androgen effects. Leydig cell dysfunction and steroidogenic failure represent key mechanisms underlying both testosterone decline and impaired spermatogenesis [26,27].

Aging is associated with progressive Leydig cell dysfunction, characterized by reduced cell number (approximately 44% lower in men aged 50–76 compared to younger men), decreased steroidogenic enzyme expression, and impaired response to LH stimulation [54,60]. At the molecular level, aging-related Leydig cell dysfunction involves oxidative stress, mitochondrial dysfunction, endoplasmic reticulum stress, and reduced expression of steroidogenic acute regulatory protein (StAR) and cytochrome P450 enzymes (CYP11A1, CYP17A1) essential for testosterone synthesis [44,61].

Obesity accelerates Leydig cell aging and dysfunction through multiple mechanisms [27]. Adipose tissue-derived inflammatory cytokines (IL-6, TNF-α) directly inhibit Leydig cell steroidogenesis [28]. Insulin resistance and hyperinsulinemia have been associated with impaired Leydig cell function [54]. Increased aromatase activity in adipose tissue converts testosterone to estradiol, creating a hyperestrogenic state that suppresses LH secretion and further reduces testosterone production [56].

Endocrine-disrupting chemicals have been associated with impaired Leydig cell steroidogenesis through multiple mechanisms. Phthalates inhibit key steroidogenic enzymes, including 3β-hydroxysteroid dehydrogenase (3β-HSD), 17β-hydroxysteroid dehydrogenase (17β-HSD), and CYP17A1, reducing testosterone synthesis [20,21]. BPA (bisphenol A) interferes with StAR protein expression and function, impairing cholesterol transport into mitochondria, the rate-limiting step in steroidogenesis [63]. Pesticides such as organophosphates and pyrethroids can directly damage Leydig cells and have been associated with disruption of steroidogenic enzyme activity [30].

Heavy metals, particularly cadmium and lead, accumulate in Leydig cells and have been associated with oxidative stress, mitochondrial dysfunction, and apoptosis [22,65]. Cadmium exposure is associated with reduced testosterone levels and impaired Leydig cell function in both animal models and human studies [66].

### 4.4. Oxidative Stress and Mitochondrial Dysfunction

Oxidative stress, defined as an imbalance between reactive oxygen species (ROS) production and antioxidant defense capacity, represents a central mechanism underlying male reproductive dysfunction [19,20]. Spermatozoa are particularly vulnerable to oxidative damage due to their high content of polyunsaturated fatty acids in the plasma membrane, limited cytoplasm (and thus limited antioxidant enzymes), and limited capacity for DNA repair [20].

Multiple environmental and lifestyle factors have been associated with oxidative stress in the male reproductive tract. Endocrine-disrupting chemicals, including BPA, phthalates, and pesticides, stimulate ROS production through mitochondrial dysfunction, NADPH oxidase activation, and disruption of antioxidant enzyme systems [20,67]. Heavy metals such as cadmium, lead, and mercury are potent inducers of oxidative stress, generating ROS through Fenton-like reactions and depleting cellular glutathione [22].

Lifestyle factors significantly contribute to reproductive oxidative stress. Obesity is associated with systemic oxidative stress and inflammation, which extend to the testis and epididymis [28]. Smoking introduces numerous oxidative compounds directly into the circulation, while also depleting antioxidant vitamins [25]. Alcohol metabolism generates acetaldehyde and ROS, contributing to testicular oxidative stress [23]. Heat exposure, whether from fever, hot baths, or occupational sources, has been associated with testicular hypoxia and oxidative stress [62].

Mitochondrial dysfunction has been associated with and is a consequence of oxidative stress in male reproduction [19]. Mitochondria are the primary source of cellular ROS, and mitochondrial dysfunction may contribute to increased ROS production [22]. Conversely, oxidative stress damages mitochondrial DNA, proteins, and lipids, further impairing mitochondrial function in a vicious cycle [44]. Sperm mitochondria are essential for energy production required for motility, capacitation, and fertilization, and mitochondrial dysfunction is strongly associated with asthenozoospermia and male infertility [19].

The consequences of oxidative stress in male reproduction are multifaceted. Lipid peroxidation damages sperm plasma membranes, impairing motility and viability [20]. Protein oxidation has been associated with disruption of enzyme function and structural proteins [19]. DNA oxidation may contribute to sperm DNA fragmentation, discussed in detail below [20]. Oxidative stress has also been associated with impaired sperm capacitation, acrosome reaction, and sperm–oocyte fusion [68].

### 4.5. Sperm DNA Fragmentation

Sperm DNA fragmentation (SDF), defined as breaks or lesions in the sperm nuclear DNA, has emerged as an important biomarker of male reproductive dysfunction and a predictor of fertility outcomes [20,25]. SDF is elevated in men with idiopathic infertility, varicocele, and exposure to environmental toxicants, and is associated with reduced natural conception rates, impaired embryo development, increased miscarriage risk, and adverse outcomes even with assisted reproductive technologies [20,25].

The mechanisms underlying SDF are diverse. Oxidative stress is the primary driver, with ROS directly attacking DNA bases and causing strand breaks [20]. Defective chromatin packaging during spermiogenesis, characterized by incomplete replacement of histones with protamines or abnormal protamine ratios, leaves DNA vulnerable to damage [20]. Apoptosis in germ cells or spermatozoa, triggered by environmental toxicants, heat, or oxidative stress, activates endonucleases that fragment DNA [23].

Environmental exposures significantly increase SDF. Smoking increases SDF by approximately 10%, likely through direct oxidative damage and depletion of seminal antioxidants [25]. Chronic alcohol consumption similarly elevates SDF [25]. Exposure to pesticides, heavy metals, and air pollution is associated with increased SDF in occupational and environmental studies [38,39]. Phthalate exposure correlates with elevated SDF in multiple human studies [69].

Lifestyle factors also influence SDF. Obesity is associated with increased SDF, likely mediated by systemic oxidative stress and inflammation [28]. Advanced paternal age is linked to increased SDF, reflecting accumulated oxidative damage and declining DNA repair capacity [4]. Heat exposure, whether from fever, hot baths, or occupational sources, transiently increases SDF [62].

The clinical significance of SDF is increasingly recognized. While conventional semen analysis assesses sperm concentration, motility, and morphology, it does not evaluate DNA integrity [45]. Men with normal semen parameters may have elevated SDF, explaining some cases of unexplained infertility [20]. SDF testing, using assays such as the sperm chromatin structure assay (SCSA), terminal deoxynucleotidyl transferase dUTP nick end labeling (TUNEL), or sperm chromatin dispersion (SCD) test, provides additional diagnostic information [20].

Interventions to reduce SDF include lifestyle modifications (smoking cessation, weight loss, heat avoidance), antioxidant supplementation, treatment of underlying conditions (varicocele repair, infection treatment), and, in severe cases, testicular sperm extraction for use in intracytoplasmic sperm injection (ICSI), as testicular sperm typically have lower SDF than ejaculated sperm [20,25].

## 5. Endocrine Disruptors: Chemical Threats to Male Reproduction

### 5.1. Bisphenol A and Analogs

Bisphenol A (BPA) is a high-production-volume industrial chemical used primarily in the manufacture of polycarbonate plastics and epoxy resins, with applications in food and beverage containers, thermal paper receipts, dental sealants, and numerous consumer products [63]. Human exposure is ubiquitous, with BPA detected in over 90% of urine samples in biomonitoring studies [20]. Concerns about BPA’s endocrine-disrupting properties have led to restrictions in some applications and substitution with structural analogs such as bisphenol F (BPF) and bisphenol S (BPS), which may have similar or even greater endocrine-disrupting activity [67].

BPA exhibits multiple mechanisms of reproductive toxicity. It acts as a weak estrogen receptor agonist, binding to both ERα and ERβ, and as an androgen receptor antagonist [20]. BPA interferes with steroidogenesis by inhibiting multiple steroidogenic enzymes, including StAR, CYP11A1, CYP17A1, and 3β-HSD, leading to reduced testosterone production in Leydig cells [20,63]. In Sertoli cells, BPA has been associated with disruption of tight junction proteins and has been associated with an impaired blood–testis barrier [20].

Human epidemiological studies have linked BPA exposure to adverse reproductive outcomes. A meta-analysis found significant negative correlations between urinary BPA concentrations and semen parameters, including sperm concentration, motility, and morphology [20]. Occupational studies of workers in BPA production facilities report elevated rates of sexual dysfunction and reduced semen quality [20]. Prenatal BPA exposure has been associated with altered anogenital distance in male infants, a marker of in utero androgen action [63].

Animal studies provide mechanistic insights into BPA’s reproductive toxicity. Rodent studies demonstrate that BPA exposure during fetal development has been associated with permanent alterations in reproductive tract development, reduced sperm production in adulthood, and increased susceptibility to reproductive disorders [20]. BPA has been associated with oxidative stress in testicular tissue, evidenced by increased lipid peroxidation, reduced antioxidant enzyme activity, and elevated sperm DNA fragmentation [20]. Epigenetic effects, including altered DNA methylation patterns in sperm, have been documented and may contribute to transgenerational effects [22].

BPF, increasingly used as a BPA substitute, exhibits similar or greater reproductive toxicity. Recent studies demonstrate that BPF has been associated with impaired spermatogenesis through UCP2-related oxidative stress and autophagy dysregulation, suppressing UCP2 expression and inactivating the AMPK/NRF2 signaling axis [67]. This establishes a comprehensive adverse outcome pathway for BPF associated with male reproductive toxicity [67].

### 5.2. Phthalates

Phthalates are a family of synthetic chemicals used as plasticizers to increase the flexibility and durability of plastics, and as solvents in personal care products, cosmetics, pharmaceuticals, and industrial applications [64]. High-molecular-weight phthalates such as di-(2-ethylhexyl) phthalate (DEHP) and di-isononyl phthalate (DiNP) are used primarily in polyvinyl chloride (PVC) products, while low-molecular-weight phthalates such as diethyl phthalate (DEP) and dibutyl phthalate (DBP) are common in personal care products [64]. Human exposure is widespread, with multiple phthalate metabolites detected in virtually all individuals in biomonitoring studies [69].

Phthalates are among the most extensively studied endocrine disruptors with respect to male reproductive health. They exert anti-androgenic effects through multiple mechanisms, including inhibition of testosterone synthesis, disruption of androgen receptor signaling, and interference with androgen-dependent developmental processes [20,21]. The “phthalate syndrome” in rodents, characterized by reduced anogenital distance, nipple retention, hypospadias, cryptorchidism, and reduced sperm production, closely resembles human testicular dysgenesis syndrome [21].

At the molecular level, phthalates have been associated with disruption of steroidogenesis by inhibiting key enzymes in the testosterone synthesis pathway. DEHP and its metabolite mono-(2-ethylhexyl) phthalate (MEHP) suppress expression of StAR, CYP11A1, CYP17A1, and 3β-HSD in Leydig cells, reducing testosterone production [20,21]. Phthalates have also been associated with oxidative stress in testicular tissue, with increased ROS production, lipid peroxidation, and depletion of antioxidant defenses [20].

Human epidemiological evidence links phthalate exposure to adverse reproductive outcomes. A systematic review and meta-analysis found that urinary concentrations of monobutyl phthalate (MBP) and monobenzyl phthalate (MBzP) were negatively correlated with semen concentration (pooled OR: 2.186 and 1.882, respectively, *p* < 0.05) [69]. DEHP in semen showed a negative correlation with sperm parameters (pooled r: −0.225, *p* < 0.05) [69]. Prenatal phthalate exposure has been associated with reduced anogenital distance in male infants, suggesting anti-androgenic effects during critical developmental windows [63].

Occupational studies provide additional evidence. Men occupationally exposed to high levels of phthalates in plastic manufacturing or other industries show reduced semen quality, altered hormone levels, and increased sperm DNA fragmentation [64]. A case-control study found significant associations between plasticizer exposure and male fertility parameters, with higher phthalate metabolite concentrations in infertile men [64].

### 5.3. Pesticides and Herbicides

Pesticides represent a diverse class of chemicals designed to control pests, weeds, and plant diseases in agricultural and non-agricultural settings. Many pesticides possess endocrine-disrupting properties and have been implicated in male reproductive dysfunction [30,40]. Major classes of concern include organophosphates, organochlorines, pyrethroids, and herbicides such as atrazine and glyphosate [30].

Organophosphate pesticides, widely used as insecticides, exert reproductive toxicity through multiple mechanisms beyond their primary action as acetylcholinesterase inhibitors [30]. They have been associated with impaired Leydig cell steroidogenesis, disruption of the HPG axis, oxidative stress, and germ cell apoptosis [30]. Occupational studies of agricultural workers exposed to organophosphates report reduced sperm concentration, motility, and increased sperm DNA fragmentation [38].

Organochlorine pesticides, including DDT, DDE, and chlordane, are persistent organic pollutants that bioaccumulate in adipose tissue and continue to pose risks despite restrictions in many countries [62]. These compounds exhibit estrogenic and anti-androgenic activities, disrupting hormone signaling and reproductive development [21]. Prenatal exposure to DDT/DDE has been associated with altered reproductive development and reduced semen quality in adulthood [21].

Pyrethroid insecticides, synthetic analogs of natural pyrethrins, are widely used in agriculture, households, and public health applications [30]. While generally considered less toxic than organophosphates or organochlorines, pyrethroids have been associated with disruption of endocrine function and impaired spermatogenesis [30]. Urinary pyrethroid metabolite concentrations have been inversely associated with semen quality parameters in human studies [30].

Herbicides present additional concerns. Atrazine, one of the most widely used herbicides globally, acts as an endocrine disruptor by increasing aromatase activity, leading to elevated estrogen levels and reduced testosterone [23]. Animal studies demonstrate that atrazine exposure has been associated with testicular damage, reduced sperm production, and altered reproductive behavior [23]. A recent study of an atrazine–mesotrione mixture demonstrated fertility decline in male rats following subchronic exposure [70].

Glyphosate, the active ingredient in Roundup and the world’s most widely used herbicide, has been associated with reproductive toxicity in animal studies, including reduced testosterone levels, altered sperm parameters, and testicular histopathological changes [30]. However, human epidemiological evidence remains limited and controversial [40].

Occupational and environmental studies provide evidence for pesticide-related reproductive dysfunction in humans. A large Tunisian study of 2122 men in infertility clinics found occupational exposure to pesticides associated with a significantly higher risk of asthenozoospermia and necrozoospermia [38]. Sperm analysis in groundnut farmers in Myanmar revealed 74% had oligozoospermia during the growing season (when pesticide exposure was highest), compared to 46% in the non-growing season [38].

### 5.4. Dioxins and Polychlorinated Biphenyls

Dioxins and polychlorinated biphenyls (PCBs) are persistent organic pollutants (POPs) that bioaccumulate in the food chain and remain detectable in human tissues decades after exposure [62]. 2,3,7,8-Tetrachlorodibenzo-p-dioxin (TCDD), the most toxic dioxin congener, is a byproduct of industrial processes and waste incineration [62]. PCBs, once widely used in electrical equipment, hydraulic fluids, and other industrial applications, were banned in many countries in the 1970s–1980s but persist in the environment [21].

These compounds exert reproductive toxicity primarily through activation of the aryl hydrocarbon receptor (AhR), a ligand-activated transcription factor that regulates expression of genes involved in xenobiotic metabolism, cell proliferation, and differentiation [62]. AhR activation can interfere with steroid hormone signaling, has been associated with disruption of reproductive development, and has been associated with impaired spermatogenesis [62].

Dioxin exposure reduces androgen action through multiple mechanisms. TCDD suppresses expression of steroidogenic enzymes in Leydig cells, reducing testosterone synthesis [62]. AhR activation interferes with androgen receptor signaling, reducing the biological effects of testosterone even when circulating levels are maintained [62]. Dioxins have also been associated with oxidative stress and apoptosis in germ cells [23].

Human evidence for dioxin-related reproductive effects comes primarily from occupational and environmental disasters. Studies of men exposed to Agent Orange (contaminated with TCDD) during the Vietnam War have reported mixed results, with some studies showing reduced semen quality and others finding no significant effects [13]. The Seveso, Italy, industrial accident in 1976, which exposed the local population to high levels of TCDD, provided an opportunity for long-term follow-up studies. Men exposed during childhood or adolescence showed reduced sperm concentration and motility in adulthood [62].

PCBs exhibit diverse endocrine-disrupting activities depending on their structure. Some PCB congeners are estrogenic, others anti-estrogenic or anti-androgenic, and some activate the AhR [21]. PCB exposure has been associated with reduced semen quality, altered hormone levels, and increased time to pregnancy in human studies [71]. A recent study demonstrated that co-exposure to polystyrene nanoplastics and PCB-126 has been associated with synergistic toxicity in human sperm, with enhanced oxidative stress and mitochondrial dysfunction [68].

Prenatal exposure to dioxins and PCBs is of particular concern, as these compounds have been associated with disruption of reproductive development during critical windows of fetal masculinization [18]. Studies have linked maternal PCB levels to reduced anogenital distance, altered reproductive hormone levels, and potentially increased risk of cryptorchidism and hypospadias in male offspring [63].

### 5.5. Limitations of the Evidence

A critical appraisal of the evidence base is essential for accurate interpretation of the associations described in this review. The majority of human data on EDC exposure and male reproductive outcomes derives from observational studies—predominantly cross-sectional designs that capture exposure and outcome at a single point in time. Such designs are inherently limited in their ability to establish temporal precedence, a fundamental requirement for causal inference. Without prospective follow-up, it remains impossible to determine with certainty whether elevated EDC exposure precedes reproductive decline or whether reverse causality or shared confounders account for the observed associations.

Confounding represents a pervasive challenge in this field. Men with higher EDC exposure may simultaneously differ from unexposed men in diet, physical activity, socioeconomic status, occupational hazards, smoking, alcohol use, and other lifestyle variables—each of which independently influences male reproductive parameters. Although many epidemiological studies attempt statistical adjustment for known confounders, residual confounding from unmeasured or poorly measured variables cannot be excluded. Furthermore, EDC exposures rarely occur in isolation; humans are exposed to complex, dynamic mixtures of chemicals whose combined effects may differ substantially from those of individual compounds studied in isolation.

The translational relevance of animal and in vitro evidence to human health outcomes must be interpreted cautiously. Rodent studies typically employ doses that substantially exceed environmentally relevant human exposures, often by one to several orders of magnitude. Pharmacokinetic differences between species—including differences in absorption, distribution, metabolism, and excretion—further limit direct extrapolation. In vitro experiments, while mechanistically informative, are conducted under artificial conditions that do not replicate the complexity of the intact organism, including systemic hormonal feedback, immune interactions, and tissue-level responses. Consequently, mechanistic pathways identified in cell culture or animal models should be regarded as hypothesis-generating rather than as direct evidence of human reproductive toxicity.

Exposure assessment methodologies introduce additional uncertainty. Urinary or serum biomarkers of EDC exposure reflect recent or short-term exposure for many compounds, yet reproductive programming may be most vulnerable during specific developmental windows—fetal life, early childhood, or puberty—that are difficult to capture retrospectively in adult cohorts. Single time-point measurements may not accurately represent cumulative or peak lifetime exposure, introducing non-differential measurement error that typically biases associations toward the null.

In light of these limitations, the associations between EDCs and male reproductive decline described throughout this review should be interpreted as suggestive of biological plausibility rather than as established causal relationships. Strengthening the evidence base will require well-designed prospective cohort studies with repeated exposure measurements across critical developmental windows, comprehensive confounder assessment, and harmonized reproductive outcome definitions. Integration of epidemiological, toxicological, and mechanistic data—applying frameworks such as the Bradford Hill criteria or systematic evidence mapping—will be essential to move from association to causation and to inform evidence-based public health policy.

### 5.6. Mechanisms of Endocrine Disruption

Endocrine-disrupting chemicals interfere with male reproductive function through diverse and often overlapping mechanisms [19,22]. Understanding these mechanisms is essential for risk assessment, biomarker development, and therapeutic intervention.

Receptor-mediated mechanisms are among the most extensively studied. EDCs can act as hormone receptor agonists, mimicking the effects of endogenous hormones by binding to and activating estrogen receptors, androgen receptors, or other nuclear receptors [20]. Conversely, EDCs can act as receptor antagonists, binding to receptors without activating them and thereby blocking the effects of endogenous hormones [19]. Some EDCs exhibit mixed agonist/antagonist activity depending on tissue context, concentration, and receptor subtype [21].

Steroidogenic enzyme inhibition represents a critical mechanism by which EDCs reduce testosterone production. Many EDCs, including phthalates, BPA, and certain pesticides, inhibit key enzymes in the steroidogenic pathway, including StAR, CYP11A1, CYP17A1, 3β-HSD, and 17β-HSD [21,63]. This enzymatic inhibition directly reduces testosterone synthesis in Leydig cells, contributing to both local (intratesticular) and systemic androgen deficiency [20].

Aromatase modulation is another important mechanism. Some EDCs increase aromatase (CYP19A1) expression or activity, enhancing conversion of testosterone to estradiol and creating a hyperestrogenic, hypoandrogenic state [23]. This is particularly relevant for pesticides such as atrazine and certain organochlorines [23]. Conversely, some EDCs inhibit aromatase, potentially disrupting the normal balance of androgens and estrogens required for spermatogenesis [21].

Oxidative stress induction is a common mechanism of EDC toxicity. Many EDCs stimulate ROS production through mitochondrial dysfunction, NADPH oxidase activation, or disruption of antioxidant defense systems [19,20]. The resulting oxidative stress damages cellular macromolecules (lipids, proteins, DNA), has been associated with impaired cell signaling, and can trigger apoptosis in germ cells, Sertoli cells, and Leydig cells [22].

Epigenetic modifications represent an emerging area of concern. EDCs can alter DNA methylation patterns, histone modifications, and microRNA expression in germ cells, potentially leading to heritable changes in gene expression [19,22]. Prenatal exposure to EDCs has been associated with epigenetic changes that persist into adulthood and may even be transmitted to subsequent generations [22]. This provides a potential mechanism for transgenerational effects of EDC exposure [3].

HPG axis disruption can occur at multiple levels. EDCs with estrogenic activity can suppress GnRH and gonadotropin secretion through negative feedback [21]. Anti-androgenic EDCs have been associated with disruption of normal androgen feedback regulation [19]. Some EDCs directly affect hypothalamic GnRH neurons or pituitary gonadotrophs, altering the pulsatile secretion patterns essential for normal reproductive function [47].

Mixture effects and low-dose effects complicate risk assessment. Humans are exposed to complex mixtures of EDCs simultaneously, and these chemicals may interact in additive, synergistic, or antagonistic ways [19,68]. Low-dose effects, where EDCs produce effects at environmentally relevant concentrations below those predicted by traditional dose-response curves, have been documented for several EDCs and challenge conventional toxicological assumptions [19].

## 6. Lifestyle and Environmental Factors

### 6.1. Obesity and Metabolic Syndrome

Obesity has emerged as one of the most significant modifiable risk factors for male reproductive dysfunction, with prevalence rates increasing dramatically worldwide over the past several decades [28]. The relationship between obesity and male infertility is multifaceted, involving hormonal, metabolic, thermal, and mechanical mechanisms [24,27].

Obesity profoundly affects the HPG axis through multiple pathways. Increased adipose tissue enhances aromatase activity, converting testosterone to estradiol and creating a hyperestrogenic state that suppresses GnRH and gonadotropin secretion [28]. This leads to secondary hypogonadism, with reduced LH levels and decreased testicular testosterone production [27]. Adipose tissue also produces leptin, which at elevated levels can suppress the reproductive axis [28]. Conversely, adiponectin, which is reduced in obesity, normally supports reproductive function [28].

Chronic low-grade inflammation associated with obesity directly impairs reproductive function. Adipose tissue in obese individuals secretes pro-inflammatory cytokines, including IL-6, TNF-α, and C-reactive protein (CRP), which suppress GnRH secretion, inhibit Leydig cell steroidogenesis, and induce oxidative stress in testicular tissue [27,54]. Insulin resistance and hyperinsulinemia, hallmarks of metabolic syndrome, further impair Leydig cell function and spermatogenesis [54].

Obesity increases scrotal temperature through increased adipose tissue insulation and reduced heat dissipation, potentially impairing spermatogenesis [24]. Mechanical factors, including increased abdominal adiposity compressing the spermatic cord and altered scrotal positioning, may contribute to varicocele formation and testicular dysfunction [24].

Epidemiological studies consistently demonstrate associations between obesity and impaired semen quality. Meta-analyses show that obese men have reduced sperm concentration, total sperm count, and progressive motility compared to normal-weight men [28]. The relationship appears dose-dependent, with more severe obesity associated with greater impairment [28]. Obesity is also associated with increased sperm DNA fragmentation and altered sperm epigenetic profiles [28].

The impact of obesity on testosterone is substantial. Cross-sectional studies show that obese men have testosterone levels approximately 30% lower than normal-weight men [53]. However, the relationship is complex and bidirectional: low testosterone promotes fat accumulation and metabolic dysfunction, while obesity suppresses testosterone production, creating a vicious cycle [28]. Weight loss through diet, exercise, or bariatric surgery can improve testosterone levels and semen parameters, though the magnitude and consistency of improvement vary [28,44].

### 6.2. Sedentary Behavior and Physical Inactivity

Physical inactivity and sedentary behavior have increased dramatically in modern societies, with potential implications for male reproductive health [23]. Prolonged sitting, particularly in occupations requiring extended periods of seated work, increases scrotal temperature and may impair spermatogenesis [23]. Professional drivers, office workers, and others with sedentary occupations show reduced semen quality in some studies [38].

Conversely, moderate physical activity appears beneficial for male reproductive health. Regular exercise improves hormonal profiles, reduces oxidative stress, enhances antioxidant defenses, and improves metabolic health [44]. Studies of men engaging in moderate-intensity exercise (3–5 h per week) show improved sperm concentration, motility, and morphology compared to sedentary men [44].

However, the relationship between exercise and reproductive health is U-shaped, with excessive exercise potentially detrimental. Elite athletes and men engaging in very high-intensity or high-volume training may experience suppression of the HPG axis, reduced testosterone levels, and impaired semen quality [23]. Endurance athletes, particularly cyclists and marathon runners, show elevated rates of reproductive dysfunction in some studies [23]. Mechanisms may include chronic elevation of cortisol, increased oxidative stress, energy deficit, and, in cyclists, mechanical trauma and heat exposure to the perineum [23].

### 6.3. Smoking and Alcohol Consumption

Cigarette smoking is a well-established risk factor for male reproductive dysfunction, with effects mediated through multiple mechanisms [25]. Tobacco smoke contains over 7000 chemicals, including numerous reproductive toxicants such as cadmium, lead, polycyclic aromatic hydrocarbons, and nicotine [62]. Smoking induces systemic oxidative stress, depletes antioxidant vitamins (particularly vitamin C and E), and introduces ROS directly into the circulation [25].

Meta-analyses demonstrate that smokers have reduced sperm concentration (approximately 15–25% lower than non-smokers), decreased motility, and increased morphological abnormalities [72]. Smoking increases sperm DNA fragmentation by approximately 10%, likely through oxidative damage [25]. The effects appear dose-dependent, with heavier smoking associated with greater impairment [25]. Smoking also affects hormone levels, with some studies showing reduced testosterone and elevated estradiol in smokers [25].

Paternal smoking may have transgenerational effects. Prenatal exposure to paternal smoking (through maternal secondhand smoke exposure) has been associated with reduced semen quality in male offspring, suggesting epigenetic or developmental programming effects [31]. Smoking cessation improves semen parameters, though the time course and magnitude of improvement vary [25].

Alcohol consumption shows a complex, dose-dependent relationship with male reproductive health [23]. Moderate alcohol consumption (1–2 drinks per day) appears to have minimal effects on semen quality or testosterone levels in most studies [23]. However, chronic heavy alcohol consumption (>5 drinks per day) is associated with reduced testosterone, impaired spermatogenesis, testicular atrophy, and sexual dysfunction [23].

Alcohol metabolism generates acetaldehyde and ROS, contributing to oxidative stress in testicular tissue [23]. Chronic alcohol consumption can directly damage Leydig cells and Sertoli cells, disrupt the HPG axis, and impair liver function, leading to altered steroid hormone metabolism [23]. Alcohol also increases aromatase activity, contributing to hyperestrogenism [23]. Studies show that chronic alcohol use increases sperm DNA fragmentation by a magnitude comparable to smoking [25].

### 6.4. Heat Exposure and Scrotal Temperature

Spermatogenesis requires a temperature approximately 2–4 °C below core body temperature, which is maintained through the scrotal location of the testes and countercurrent heat exchange in the pampiniform plexus [62]. Elevation of testicular temperature impairs spermatogenesis through multiple mechanisms, including induction of hypoxia, oxidative stress, germ cell apoptosis, and disruption of Sertoli cell function [62].

Occupational heat exposure represents a significant risk factor for male infertility. Men working in high-temperature environments (bakers, welders, foundry workers, professional drivers) show reduced semen quality in multiple studies [38]. Professional drivers, who experience both heat exposure and prolonged sitting, are at particularly high risk [38].

Lifestyle factors that increase scrotal temperature include hot baths, saunas, laptop computer use on the lap, and tight-fitting underwear [23]. Frequent sauna use (>15 min, >2 times per week) has been associated with reduced sperm concentration and motility, though effects are typically reversible with cessation [23]. Laptop computer use directly on the lap increases scrotal temperature by 2–3 °C and may impair semen quality with chronic use [23].

Fever from illness can transiently impair spermatogenesis, with effects typically appearing 2–3 months later (reflecting the duration of spermatogenesis) and recovering within several months [62]. Varicocele, a common cause of male infertility, impairs spermatogenesis partly through increased testicular temperature due to impaired venous drainage [73].

At the molecular level, heat stress induces expression of heat shock proteins, increases ROS production, activates apoptotic pathways, and disrupts the blood–testis barrier [62]. Heat exposure suppresses expression of steroidogenic enzymes, reducing intratesticular testosterone concentrations [74]. Global warming and increasing environmental temperatures may represent an emerging threat to male reproductive health at the population level [74].

### 6.5. Sleep Disorders and Chronic Stress

Sleep is essential for reproductive health, with testosterone secretion following a circadian rhythm characterized by peak levels in the early morning and nadir in the evening [49]. Sleep deprivation and sleep disorders disrupt this rhythm and can impair reproductive function [23]. Studies show that men sleeping less than 6 h per night have reduced testosterone levels and impaired semen quality compared to those sleeping 7–8 h [23].

Obstructive sleep apnea (OSA), characterized by repeated episodes of upper airway obstruction during sleep, is associated with hypogonadism and sexual dysfunction [23]. OSA causes intermittent hypoxia, sleep fragmentation, and activation of the sympathetic nervous system, all of which can suppress the HPG axis and reduce testosterone production [23]. Treatment of OSA with continuous positive airway pressure (CPAP) can improve testosterone levels in some patients [23].

Chronic psychological stress activates the hypothalamic–pituitary–adrenal (HPA) axis, leading to elevated cortisol levels [3]. Cortisol can suppress GnRH secretion, inhibit Leydig cell steroidogenesis, and impair spermatogenesis [3]. Chronic stress is associated with reduced testosterone levels, impaired semen quality, and sexual dysfunction in multiple studies [3]. Stress management interventions, including cognitive–behavioral therapy and mindfulness-based stress reduction, may improve reproductive outcomes in some men [3].

### 6.6. Air Pollution

Air pollution has emerged as a significant environmental threat to male reproductive health, with growing evidence linking exposure to particulate matter (PM), nitrogen oxides (NOx), ozone (O_3_), and other pollutants to impaired semen quality and reduced testosterone levels [39,75]. The mechanisms involve systemic inflammation, oxidative stress, endocrine disruption, and direct toxicity to reproductive tissues [39].

Particulate matter, particularly PM2.5 (particles < 2.5 μm in diameter), can penetrate deep into the lungs and enter the systemic circulation, inducing inflammation and oxidative stress [39]. PM contains numerous toxic components, including heavy metals, polycyclic aromatic hydrocarbons (PAHs), and endocrine-disrupting chemicals [62]. Studies show associations between PM2.5 exposure and reduced sperm concentration, motility, and morphology [39].

Traffic-related air pollution, characterized by elevated levels of nitrogen dioxide (NO_2_), carbon monoxide (CO), and diesel exhaust particles, has been linked to male reproductive dysfunction in multiple studies [62]. Men living in areas with high traffic density or near major roadways show reduced semen quality compared to those in less polluted areas [39]. Occupational exposure to traffic exhaust, as experienced by professional drivers, taxi drivers, and traffic police, is associated with impaired reproductive parameters [38].

PAHs, formed during the incomplete combustion of organic materials and present in vehicle exhaust, tobacco smoke, and grilled foods, are reproductive toxicants [62]. PAH exposure is associated with reduced sperm concentration, increased sperm DNA fragmentation, and altered sperm epigenetic profiles [62]. PAHs can bind to the AhR and disrupt steroid hormone signaling [62].

Ozone, a secondary pollutant formed through photochemical reactions, is a potent oxidant that can induce systemic oxidative stress [39]. Studies have linked ozone exposure to reduced semen quality, though findings are less consistent than for particulate matter [39].

### 6.7. Heavy Metals

Heavy metals, including lead, cadmium, mercury, arsenic, and aluminum, are ubiquitous environmental contaminants with well-documented reproductive toxicity [22,30]. These metals bioaccumulate in tissues, including the testis, and exert toxicity through oxidative stress, disruption of cellular signaling, interference with essential metal homeostasis, and direct damage to cellular structures [22].

Lead exposure, historically from leaded gasoline and paint but now primarily from occupational sources, contaminated water, and legacy environmental contamination, impairs male reproductive function [62]. Lead interferes with the HPG axis, reduces testosterone synthesis, impairs spermatogenesis, and increases sperm DNA fragmentation [30]. Occupational studies show dose-dependent relationships between blood lead levels and semen quality impairment [30].

Cadmium, present in tobacco smoke, contaminated food (particularly shellfish and organ meats), and industrial emissions, is a potent reproductive toxicant [62]. Cadmium accumulates in the testis and directly damages Leydig cells and Sertoli cells [65]. It induces oxidative stress, disrupts inter-Sertoli cell tight junctions, and impairs steroidogenesis [66]. Smoking is a major source of cadmium exposure, contributing to smoking-related reproductive dysfunction [62].

Mercury, particularly methylmercury from contaminated fish, crosses the blood–testis barrier and impairs spermatogenesis [62]. Mercury induces oxidative stress, disrupts microtubule assembly in developing spermatids, and can cause germ cell apoptosis [62]. Occupational exposure to mercury vapor in dental workers and industrial settings has been associated with reduced semen quality [30].

Arsenic, present in contaminated drinking water in many regions worldwide, exhibits reproductive toxicity through oxidative stress, disruption of steroid hormone signaling, and epigenetic modifications [22]. Studies in arsenic-endemic areas show associations between arsenic exposure and reduced semen quality, altered hormone levels, and increased infertility [22].

A recent study examining the interaction of toxic metal exposure and oxidative balance score on semen quality found that metals like lead, mercury, and barium negatively correlate with semen quality parameters, with significant interactions between oxidative balance and metal toxicity [76]. This highlights the importance of antioxidant status in modulating metal-induced reproductive toxicity.

### 6.8. Microplastics and Emerging Contaminants

Microplastics, defined as plastic particles less than 5 mm in diameter, have emerged as a ubiquitous environmental contaminant with potential implications for human health, including reproductive function [68]. These particles result from the degradation of larger plastic items and are present in air, water, food, and consumer products [68]. Nanoplastics (particles < 1 μm) can penetrate cellular membranes and potentially cross the blood–testis barrier [68].

Recent studies have detected microplastics in human tissues, including the placenta, blood, and potentially the testis [68]. The reproductive toxicity of microplastics may result from physical effects (particle-induced inflammation and tissue damage), chemical effects (leaching of plastic additives including phthalates, BPA, and other EDCs), and carrier effects (adsorption of other environmental contaminants onto plastic surfaces) [68].

Animal studies demonstrate that microplastic exposure can impair spermatogenesis, reduce sperm quality, and alter reproductive hormone levels [68]. A recent study showed that co-exposure to polystyrene nanoplastics and PCB-126 induces synergistic toxicity in human sperm, with enhanced oxidative stress, mitochondrial dysfunction, and impaired capacitation and acrosome reaction [68]. This suggests that microplastics may amplify the toxicity of other environmental contaminants.

Per- and polyfluoroalkyl substances (PFAS), synthetic chemicals used in non-stick cookware, water-repellent fabrics, food packaging, and firefighting foams, are persistent environmental contaminants with endocrine-disrupting properties [40]. PFAS bioaccumulate in human tissues and have been detected in blood samples from virtually all individuals in biomonitoring studies [40]. Some PFAS compounds have been associated with reduced semen quality, altered reproductive hormone levels, and increased time to pregnancy in human studies [40].

Pharmaceutical residues in drinking water and the environment represent another emerging concern. Hormones from contraceptives and hormone replacement therapy, as well as other pharmaceuticals with endocrine activity, can enter water supplies through wastewater treatment plants [66]. While concentrations are typically low, chronic low-dose exposure during critical developmental windows may have effects, particularly for compounds with high potency [66].

## 7. Clinical Implications and Therapeutic Perspectives

### 7.1. Diagnostic Approaches

The evaluation of male reproductive dysfunction requires a comprehensive approach integrating clinical history, physical examination, hormonal assessment, and semen analysis [4]. Given the multifactorial etiology of declining sperm count and testosterone levels, diagnostic evaluation should assess both reproductive parameters and potential contributing factors [73].

Semen analysis remains the cornerstone of male fertility evaluation. The WHO Laboratory Manual for the Examination and Processing of Human Semen (6th edition, 2021) provides standardized methods and reference values [45]. Key parameters include semen volume, sperm concentration, total sperm count, progressive motility, total motility, and morphology [45]. However, conventional semen analysis has limitations, including high intra-individual variability, requiring at least two samples collected 2–4 weeks apart [45].

Advanced semen tests provide additional diagnostic information. Sperm DNA fragmentation testing, using assays such as TUNEL, SCSA, or SCD, identifies men with elevated DNA damage who may have reduced fertility potential despite normal conventional parameters [20]. Oxidative stress assessment in semen, measuring ROS levels or markers of lipid peroxidation, can identify men who may benefit from antioxidant therapy [20]. Sperm chromatin maturity tests assess the completeness of the histone-to-protamine transition during spermiogenesis [20].

Hormonal evaluation should include measurement of total testosterone (preferably on a morning sample), LH, FSH, and in some cases, estradiol, prolactin, and SHBG [53]. Free or bioavailable testosterone may be more clinically relevant than total testosterone, particularly in older men or those with altered SHBG levels [54]. Diagnosis of hypogonadism requires both biochemical evidence (consistently low testosterone on repeat testing) and clinical symptoms [53].

Environmental and occupational history is essential, given the role of EDCs and other exposures in male reproductive dysfunction. Clinicians should inquire about occupational exposures (pesticides, heavy metals, heat, solvents), use of plastics and personal care products, dietary habits (particularly consumption of fish potentially contaminated with mercury or PCBs), smoking, alcohol use, and medication use [38].

Genetic testing may be indicated in men with severe oligozoospermia or azoospermia. Karyotype analysis identifies chromosomal abnormalities such as Klinefelter syndrome (47, XXY), while Y chromosome microdeletion testing detects deletions in the azoospermia factor (AZF) regions [73]. Cystic fibrosis transmembrane conductance regulator (CFTR) gene mutations should be tested in men with obstructive azoospermia or congenital bilateral absence of the vas deferens [73].

### 7.2. Lifestyle Interventions

Lifestyle modification represents the first-line approach for many men with reproductive dysfunction, particularly those with modifiable risk factors [44]. Evidence supports multiple lifestyle interventions, though the magnitude of benefit varies among individuals [43].

Weight loss in obese men can improve testosterone levels and semen parameters. Meta-analyses show that weight loss through diet and exercise increases testosterone by approximately 3–4 nmol/L and improves sperm concentration and motility [28]. Bariatric surgery in severely obese men produces more substantial improvements in testosterone (increases of 8–10 nmol/L) and may improve semen quality, though data are limited [28]. Weight loss interventions should target a gradual, sustainable reduction through caloric restriction and increased physical activity [44]. Smoking cessation improves semen parameters and reduces sperm DNA fragmentation, with benefits typically apparent within 3–6 months [25]. Comprehensive smoking cessation programs, including behavioral counseling and pharmacotherapy (nicotine replacement, varenicline, or bupropion), should be offered to all men who smoke [25].

Alcohol moderation is recommended, with guidelines suggesting no more than 1–2 drinks per day [23]. Men with heavy alcohol consumption should be counseled on reduction or abstinence, with referral to addiction services if needed [23].

Heat avoidance includes recommendations to avoid hot baths, saunas, prolonged laptop use on the lap, and tight-fitting underwear [23]. Occupational heat exposure may require workplace modifications or job changes in severe cases [38].

Sleep optimization includes maintaining regular sleep schedules, ensuring 7–8 h of sleep per night, and evaluation and treatment of sleep disorders such as obstructive sleep apnea [23].

Stress management through cognitive–behavioral therapy, mindfulness-based stress reduction, or other psychological interventions may benefit men with stress-related reproductive dysfunction [3].

Dietary optimization should emphasize a Mediterranean-style diet rich in fruits, vegetables, whole grains, fish, nuts, and olive oil, which has been associated with improved semen quality and testosterone levels [44]. Specific nutrients of interest include omega-3 fatty acids, antioxidants (vitamins C and E, selenium, zinc), and folate [44].

### 7.3. Antioxidant Therapy

Given the central role of oxidative stress in male reproductive dysfunction, antioxidant supplementation has been extensively investigated as a therapeutic intervention [20]. Multiple antioxidants have been studied, including vitamins C and E, selenium, zinc, coenzyme Q10, L-carnitine, N-acetylcysteine, and lycopene [44].

Meta-analyses of randomized controlled trials show that antioxidant supplementation improves semen parameters, particularly sperm concentration and motility, and may increase pregnancy rates in couples undergoing fertility treatment [44]. However, the evidence is heterogeneous, with substantial variability in the specific antioxidants used, dosages, treatment duration, and patient populations [44].

Vitamin C (ascorbic acid) is a water-soluble antioxidant present in high concentrations in seminal plasma, where it protects sperm from oxidative damage [44]. Supplementation with vitamin C (500–1000 mg daily) has been shown to improve sperm quality in some studies [44].

Vitamin E (α-tocopherol) is a lipid-soluble antioxidant that protects sperm membrane lipids from peroxidation [44]. Doses of 400–600 IU daily have been used in clinical trials, with some showing improvements in sperm motility and DNA integrity [44].

Selenium is an essential trace element and cofactor for glutathione peroxidase, a key antioxidant enzyme [44]. Selenium supplementation (100–200 μg daily) has been associated with improved sperm motility and morphology in selenium-deficient men [44].

Zinc is essential for spermatogenesis, testosterone synthesis, and antioxidant defense [44]. Zinc supplementation (25–50 mg daily) may improve semen parameters in zinc-deficient men, though excessive zinc can be harmful [44].

Coenzyme Q10 (ubiquinone) is a lipid-soluble antioxidant and essential component of the mitochondrial electron transport chain [44]. Supplementation with CoQ10 (200–300 mg daily) has shown promise in improving sperm concentration, motility, and DNA integrity, particularly in men with idiopathic infertility [44].

L-carnitine and L-acetyl-carnitine are involved in fatty acid metabolism and energy production in sperm [44]. Supplementation (1–3 g daily) has been associated with improved sperm motility and concentration in some studies [44].

Combination antioxidant formulations containing multiple antioxidants may be more effective than single agents, based on the rationale that different antioxidants work synergistically and target different cellular compartments [44]. However, optimal combinations and dosages remain to be established [44].

Important caveats regarding antioxidant therapy include the need for individualized treatment based on evidence of oxidative stress (rather than empiric supplementation in all men), potential risks of excessive antioxidant intake (which can paradoxically induce oxidative stress), and the importance of addressing underlying causes of oxidative stress (smoking, obesity, environmental exposures) rather than relying solely on supplementation [20].

### 7.4. Endocrine Treatment

Testosterone replacement therapy (TRT) is indicated for men with symptomatic hypogonadism (low testosterone with associated symptoms) who are not seeking fertility [53]. TRT improves sexual function, mood, energy, muscle mass, bone density, and metabolic parameters in hypogonadal men [53]. However, TRT suppresses gonadotropin secretion and spermatogenesis, making it contraindicated in men desiring fertility [53].

For hypogonadal men seeking fertility, alternative approaches are required. Selective estrogen receptor modulators (SERMs), particularly clomiphene citrate and enclomiphene, block estrogen receptors in the hypothalamus and pituitary, reducing negative feedback and increasing endogenous gonadotropin and testosterone production [53]. Clomiphene (25–50 mg daily or every other day) can increase testosterone levels while maintaining or improving spermatogenesis [53]. This approach is particularly useful in men with secondary hypogonadism due to obesity or other causes of HPG axis suppression [53].

Aromatase inhibitors, such as anastrozole or letrozole, reduce the conversion of testosterone to estradiol, lowering estrogen levels and increasing testosterone through reduced negative feedback [53]. These agents may be useful in men with elevated estradiol levels, particularly obese men with increased aromatase activity in adipose tissue [28]. However, evidence for efficacy in improving fertility outcomes is limited [53].

Human chorionic gonadotropin (hCG), which mimics LH, directly stimulates Leydig cells to produce testosterone [53]. hCG therapy (1000–2000 IU subcutaneously 2–3 times per week) can restore testosterone levels and spermatogenesis in men with hypogonadotropic hypogonadism [53]. Combined therapy with hCG and recombinant FSH may be necessary in men with severe gonadotropin deficiency [53].

Varicocele repair, through surgical ligation or percutaneous embolization of dilated spermatic veins, improves semen parameters and may increase pregnancy rates in men with clinical varicocele and abnormal semen analysis [73]. The mechanism involves reduction of testicular temperature, improved blood flow, and decreased oxidative stress [73].

### 7.5. Assisted Reproductive Technologies

For couples who do not achieve pregnancy through lifestyle modifications and medical management, assisted reproductive technologies (ART) offer additional options [73]. The choice of ART depends on semen parameters, female factors, duration of infertility, and patient preferences [73].

Intrauterine insemination (IUI) involves placing processed sperm directly into the uterine cavity, bypassing cervical factors and concentrating motile sperm near the fallopian tubes [73]. IUI is appropriate for couples with mild male factor infertility (reduced sperm count or motility but adequate numbers of motile sperm after processing), unexplained infertility, or cervical factor infertility [73].

In vitro fertilization (IVF) involves ovarian stimulation, oocyte retrieval, fertilization in the laboratory, and embryo transfer [73]. Conventional IVF, where oocytes and sperm are incubated together, requires adequate numbers of motile sperm [73]. IVF is appropriate for couples with moderate male factor infertility, tubal factor infertility, or failed IUI cycles [73].

Intracytoplasmic sperm injection (ICSI) involves direct injection of a single sperm into an oocyte, bypassing natural fertilization barriers [73]. ICSI has revolutionized the treatment of severe male factor infertility, allowing men with very low sperm counts, poor motility, or high rates of abnormal morphology to achieve biological fatherhood [73]. ICSI is also used in cases of high sperm DNA fragmentation, previous fertilization failure with conventional IVF, or when using surgically retrieved sperm [73].

Surgical sperm retrieval techniques, including testicular sperm extraction (TESE), microdissection TESE (micro-TESE), and percutaneous epididymal sperm aspiration (PESA), allow men with azoospermia to father biological children [73]. Micro-TESE, which uses an operating microscope to identify seminiferous tubules most likely to contain sperm, has the highest success rates in men with non-obstructive azoospermia [73].

Preimplantation genetic testing (PGT) can be performed on embryos created through IVF/ICSI to screen for chromosomal abnormalities (PGT-A) or specific genetic conditions (PGT-M) [73]. This may be particularly relevant for couples where the male partner has genetic factors contributing to infertility or carries genetic mutations [73].

Important considerations with ART include the potential for transmission of genetic or epigenetic abnormalities associated with severe male factor infertility, the psychological and financial burden of treatment, and the need for comprehensive counseling regarding success rates, risks, and alternatives, including donor sperm or adoption [73].

## 8. Conclusions

The global decline in sperm count and testosterone levels represents one of the most significant and underappreciated public health challenges of the twenty-first century. The evidence, synthesized in this review, demonstrates that male reproductive parameters have deteriorated substantially over the past five decades, with sperm concentration declining by more than 50% and testosterone levels decreasing by approximately 1% per year in an age-independent manner across multiple populations [2,8,9,10,11,12]. This phenomenon transcends geographic boundaries, affecting men in Western and non-Western countries alike, though with regional variations in magnitude and temporal patterns [8,34].

The etiology of this crisis is multifactorial and complex, involving intricate interactions between genetic susceptibility, environmental exposures, and lifestyle factors. Endocrine-disrupting chemicals—including bisphenol A, phthalates, pesticides, dioxins, and polychlorinated biphenyls—emerge as prime suspects, with robust mechanistic evidence demonstrating their capacity to have been associated with disruption of the hypothalamic–pituitary–gonadal axis, impaired steroidogenesis, oxidative stress, and epigenetic modifications [18,19,20,21,22]. The ubiquity of human exposure to these chemicals, combined with their persistence in the environment and potential for transgenerational effects, raises profound concerns about long-term impacts on human reproductive capacity [19,22].

Lifestyle factors, particularly the obesity epidemic, sedentary behavior, smoking, alcohol consumption, and chronic stress, contribute synergistically to reproductive dysfunction [3,23,25,27,28]. The convergence of these factors in modern industrialized societies may explain the accelerating decline in sperm count observed post-2000 [8]. Emerging environmental threats, including air pollution, microplastics, and climate change-related heat exposure, add additional layers of complexity and concern [39,68,74].

At the cellular and molecular level, the mechanisms underlying male reproductive decline involve disruption of the HPG axis, Leydig cell senescence and dysfunction, Sertoli cell impairment, oxidative stress, mitochondrial dysfunction, DNA damage, and epigenetic modifications [26,27,28,29,30]. These pathophysiological processes are interconnected, often forming vicious cycles that amplify reproductive dysfunction. Understanding these mechanisms is essential for developing targeted therapeutic interventions and identifying biomarkers for early detection and monitoring.

The clinical implications extend far beyond fertility. Low testosterone is associated with increased risks of metabolic syndrome, type 2 diabetes, cardiovascular disease, osteoporosis, sarcopenia, cognitive decline, depression, and all have been associated with mortality [54,55,56]. The population-level decline in testosterone may therefore contribute to the rising burden of chronic diseases in aging populations. Moreover, the potential for epigenetic transmission of reproductive dysfunction to future generations raises the specter of compounding effects across multiple generations [3,22].

Future research priorities include longitudinal cohort studies with comprehensive exposure assessment, mechanistic investigations using multi-omics approaches (transcriptomics, proteomics, metabolomics, epigenomics), mixture toxicology studies, transgenerational analyses, intervention trials for antioxidant and lifestyle therapies, biomarker development for early detection, and health disparities research to address environmental justice concerns.

Public health and policy implications are profound. Prevention through environmental policy represents the most effective long-term strategy, encompassing stricter regulation of endocrine-disrupting chemicals (with attention to cumulative and mixture effects), reduction of plastic use and improved waste management, sustainable agricultural practices to reduce pesticide use, air quality improvements, occupational health protections for workers exposed to reproductive toxicants, and public education campaigns on modifiable risk factors such as obesity, smoking, and heat exposure.

Clinical practice must evolve to address this crisis: clinicians should routinely assess environmental and occupational exposures, provide evidence-based lifestyle counseling, consider advanced semen testing (DNA fragmentation, oxidative stress markers), adopt a preventive approach, and recognize that current WHO ‘normal’ semen parameters may not represent optimal fertility potential given population-level declines.

Societal implications warrant serious consideration. Declining fertility rates in many countries, while multifactorial, may be partly attributable to reduced biological reproductive capacity. This has implications for population demographics, economic productivity, and social structures. The potential for widening health disparities, with disadvantaged populations experiencing greater exposure to reproductive toxicants, raises issues of environmental justice. Notably, the global TFR has declined from 4.84 in 1950 to 2.23 in 2021, with projections of further decline below replacement level in most world regions by 2100. Male factor infertility, contributing to approximately 50% of infertile couples, represents a significant biological component of this trend. Evidence of declining unassisted pregnancy rates in high-income countries suggests that impaired male fecundity—driven by the environmental and lifestyle factors reviewed herein—may be amplifying the demographic transition beyond what can be explained by behavioral change alone [14,17].

In conclusion, the global decline in sperm count and testosterone levels represents a complex, multifactorial public health crisis requiring urgent, coordinated action across multiple sectors. While individual clinical interventions can help affected men, addressing the root cause associated with environmental policy, lifestyle modification at the population level, and continued research into mechanisms and interventions is essential. The reproductive health of future generations depends on actions taken today to reduce environmental contamination, promote healthy lifestyles, and develop effective therapeutic strategies. This is not merely a medical issue but a societal imperative that demands the attention of policymakers, public health officials, clinicians, researchers, and the public.

## Data Availability

No new data were created or analyzed in this study. Data sharing is not applicable to this article.

## Nomenclature

**Sperm concentration**	Number of spermatozoa per milliliter of semen. WHO 6th edition lower reference limit (5th percentile): 16 million/mL.
**Total sperm count**	Total number of spermatozoa in the entire ejaculate. WHO 6th edition lower reference limit: 39 million.
**Progressive motility**	Percentage of spermatozoa moving actively forward. WHO 6th edition lower reference limit: 30%.
**Sperm DNA fragmentation (SDF)**	Breaks or lesions in sperm nuclear DNA, assessed by TUNEL, SCSA, or SCD assays.
**Hypogonadism**	Clinical syndrome resulting from failure of the testis to produce physiological concentrations of testosterone and/or a normal number of spermatozoa.
**Secular trend**	A long-term change in a population parameter over time, independent of age.
**Endocrine-disrupting chemical (EDC)**	An exogenous substance or mixture that alters function(s) of the endocrine system and consequently causes adverse health effects.
**Testicular dysgenesis syndrome (TDS)**	A hypothesis proposing that cryptorchidism, hypospadias, reduced semen quality, and testicular cancer share a common etiology in disrupted fetal testicular development.
**Oxidative stress**	An imbalance between production of reactive oxygen species (ROS) and antioxidant defenses, leading to cellular damage.
**Hypothalamic–pituitary–gonadal (HPG) axis**	The neuroendocrine system regulating male reproduction, consisting of hypothalamic GnRH, pituitary LH and FSH, and gonadal testosterone secretion.
